# Application of CRISPR screen in mechanistic studies of tumor development, tumor drug resistance, and tumor immunotherapy

**DOI:** 10.3389/fcell.2023.1220376

**Published:** 2023-06-23

**Authors:** Min Li, Jin Sun, Guohai Shi

**Affiliations:** ^1^ Key Laboratory of Nutrition and Metabolism, Institute for Nutritional Sciences, Shanghai Institutes for Biological Sciences, Chinese Academy of Sciences and Graduate School of Chinese Academy of Sciences, Shanghai, China; ^2^ Department of Urology, Fudan University Shanghai Cancer Center, Shanghai, China; ^3^ Department of Oncology, Shanghai Medical College, Shanghai, China

**Keywords:** CRISPR screen, tumor development and progression, tumor microenvironment (TME), gene edited, tumor therapeutic

## Abstract

Tumor is one of the biggest threats to human health. Though tumor therapy has been dramatically advanced by the progress of technology and research in recent decades, it is still far from expectations. Thus, it is of great significance to explore the mechanisms of tumor growth, metastasis, and resistance. Screen based on Clustered Regularly Interspaced Short Palindromic Repeats (CRISPR)-CRISPR-associated protein (Cas) 9 gene editing technology are powerful tools for exploring the abovementioned facets. This review summarizes the recent screen performed in cancer cells and immune cells in the tumor microenvironment. The screens in cancer cells mainly focus on exploring the mechanisms underlying cancer cells’ growth, metastasis, and how cancer cells escape from the FDA approved drugs or immunotherapy. And the studies in tumor-associated immune cells are primarily aimed at identifying signaling pathways that can enhance the anti-tumor function of cytotoxic T lymphocytes (CTLs), CAR-T cells, and macrophages. Moreover, we discuss the limitations, merits of the CRISPR screen, and further its future application in tumor studies. Importantly, recent advances in high throughput tumor related CRISPR screen have deeply contributed to new concepts and mechanisms underlying tumor development, tumor drug resistance, and tumor immune therapy, all of which will eventually potentiate the clinical therapy for tumor patients.

## 1 Introduction

Cancer is a leading cause of death and an important obstacle to improve life expectancy. Worldwide, there are estimated to be nearly 19.3 million new cancer cases and almost 10 million cancer related deaths in 2020 (Sung et al., 2021). It is also estimated that the global cancer burden will reach 28.4 million cases in 2040, an increase of 47% over 2020 (Sung et al., 2021). Tumor is a kind of disease with complex pathological mechanism that endangers human health severely. Fundamentally, it is a genomic disease, caused by genome alteration, including DNA mutations that activate oncogenes and inactivate tumor suppressors, as well as dysregulation of epigenomes that coordinate normal gene expression (Katti et al., 2022). Up to now, tumor treatment still faced a severe situation. The discovery of new targets for safe and highly efficient drugs is still the focus of basic and clinical research. For patients with early-stage tumors, surgical resection is an effective treatment. However, due to the difficult diagnosis and rapid progress, most patients are diagnosed in the progression stage at their initial visits, missing the opportunity for radical surgical resection, and can only accept local treatment or systematic treatment (Sangro et al., 2021; Llovet et al., 2022). Given the progress in the prevention and treatment of tumors, the overall survival of tumor patients has improved, with some patients being cured. The main reason for the progress in cancer therapy is the understanding of the underlying tumor biology, which leads to the development of small molecules and antibodies targeting key proteins in oncogenic signaling pathways. Most advance and successful examples include targeting BCR-ABL with imatinib in chronic myeloid leukemia, inhibiting EGFR with cetuximab in colorectal cancer, antagonizing BRAF with vemurafenib in melanoma, and inhibiting multiple tyrosine kinases with lenvatinib in hepatocellular carcinoma (Cunningham et al., 2004; Baccarani et al., 2009; Shaitelman et al., 2015; Kudo et al., 2018). Moreover, immune checkpoint blocking (ICB) antibodies against programmed death 1/programmed death-ligand 1 (PD-1/PD-L1) and cytotoxic T-lymphocyte-associated protein4 (CTLA-4) are designed to reactivate tumor-specific T cells, which have demonstrated effectiveness against a large number of cancer types, including melanoma, non-small-cell lung cancer, and renal cancer (Hodi et al., 2010; Chen and Mellman, 2017; Hellmann et al., 2018; Motzer et al., 2018). The ICB therapy is a main pillar of innovative cancer therapy, and its monotherapy or combined therapy with target or chemotherapeutic drugs have become popular therapeutic strategies (Sangro et al., 2021; Cortiula et al., 2022). Although tremendous progress has been made in ICB therapy, the proportion of tumor patients who have benefited from such therapy is still not high. For melanomas, only 17%–26% of patients respond to anti-PD-L1 therapy and only 2%–6% respond to anti-CTLA-4 therapy (Hodi et al., 2010; Page et al., 2014). While the response rate of liver cancer patients to PD1 antibody (nivolumab) is only 14.3% (Villanueva, 2019). PD-L1 antibody (atezolizumab) combined with VEGF antibody (bevacizumab) can improve the treatment response rate of liver cancer patients to 27.3%, still not enough (Galle et al., 2021). Thus, it is still a long way to find more therapeutic targets for developing personalized tumor treatment schemes that benefit specific patients.

The tumor microenvironment (TME) is one of the most important barriers for cancer therapy. The immune structure of the TME, commonly known as the “immune environment”, has been proven to convey reliable prognosis and predictive information for various solid tumors (Bruni et al., 2020; Petroni et al., 2022). Specifically, high intratumorally levels of CD8^+^ T cells (also known as cytotoxic T lymphocytes, CTLs), helper T cell 1 (Th1)-polarized CD4^+^ T cells, natural killer (NK) cells, mature dendritic cells (mDCs), and pro-inflammatory M1 like tumor associated macrophages (TAMs) are generally but not invariably associated with improved disease outcomes in different patient cohorts. In contrast, abundant tumor infiltration by immunosuppressive CD4^+^ CD25^+^ Foxp3^+^ regulatory T (Treg) cells, exhaustion CD8^+^ T cells (expressing PD1, Tim3, and/or Lag3), immature or tolerant DCs, anti-inflammatory M2 like TAMs, and/or myeloid-derived suppressor cells (MDSCs) are generally associated with limited sensitivity to therapy (Bruni et al., 2020; Petroni et al., 2022). Although these observations cannot be universally applied to all solid tumors, for example, high levels of intratumorally CD8^+^ T cells are associated with a poor prognosis in patients with renal cell carcinoma and metabolic activation of intrahepatic CD8^+^ T cells and NKT cells has been shown to promote NASH (Nonalcoholic Fatty Liver Disease) and HCC (hepatocellular carcinoma) via cross-talk with hepatocytes (Vesely et al., 2011; Wolf et al., 2014; Bruni et al., 2020; Petroni et al., 2022). Exploring the composition, subgroup, and function of immune cell subsets in the TME can not only help us understand the tumor microenvironment but also provide necessary guarantees for further exploring the heterogeneity of tumors and developing personalized therapeutic drugs.

The Clustered regularly interspaced short palindromic repeats (CRISPR)-CRISPR associated protein 9 system is a component of the adaptive immune system of ancient bacteria (Barrangou et al., 2007). In the past three decades, some scientists have contributed to understanding CRISPR biology and developing CRISPR gene editing technology, including establishing programmable DNA editing in mammalian cells (Cho et al., 2013; Cong et al., 2013; Mali et al., 2013). Since then, CRISPR gene editing technology has become a useful tool for programmable gene modification in almost all cell types, especially in exploring gene functions in tumor growth. Notably, the genome-wide CRISPR-Cas9-mediated screen is a powerful tool for identifying genes responsible for diverse phenotypes. CRISPR technology as an efficient tool for large-scale genetic screen has enabled great advances in cancer research, including those aiming to discover and validate therapeutic targets (Yin et al., 2019).

In this review, we first introduce the genome editing technologies, and then focus on the application of the CRISPR system in studies of cancer cells and the cell subpopulations in TME. At last, we also prospect the future application of the CRISPR screen in clinical tumor therapy and potential drug exploration. Significantly, here we have comprehensively summarized current cancer studies using CRISPR screen technology and their achievements, which provides fruitful information helping to understand the research models and trends in this field.

## 2 The introduction of genome editing technology

Nearly a decade ago, the zinc-finger nucleases (ZFNs) technology was developed as the first practical tool for genome editing (Urnov et al., 2010). Each zinc-finger protein consists of three or more zinc-finger domains, each of which interacts with a 3 bp DNA sequence, which has high specificity (Urnov et al., 2010). The efficiency of genome editing by ZFNs can be high, but a fairly complicated process of protein engineering is required to target specific DNA sequence (Yin et al., 2019). In one trial, ZFNs was used to disrupt the CCR5 gene in T cells isolated from HIV patients, which were subsequently expanded and reinjected into patients to generate an HIV-resistant autologous T cell pool (Perez et al., 2008). Simultaneously, another gene editing technology using transcription activators like effector nucleases (TALENs) were developed for efficient gene editing (Joung and Sander, 2013). TALENs contains a FokI nuclease domain fused to a DNA binding domain, which is engineered with a series of highly conserved 34 aa-repeats derived from transcription activator like effectors (TALEs) produced by different species of *Xanthomonas*. Each DNA-binding TALE repeat binds to an individual base of the four, allowing any sequences to be targeted by TALENs. Both ZFN and TALENs can introduce DNA double-strand breaks (DSBs), which are repaired by non-homologous end-joining (NHEJ) or homology directed repair (HDR), so that DNA sequences can be deleted or inserted (Joung and Sander, 2013). Since new ZFN or TALEN proteins must be engineered for each new target site, and ZFN can only target limited number of genome sites, such design constraints have restricted the application of these two technologies.

The CRISPR-Cas9 system was first applied in genome editing by the laboratories of Emmanuelle Charpentier, Jennifer Doudna, and Feng Zhang. CRISPR-Cas9 system consists of crRNA: trancrRNA duplex and Cas9 endonuclease. For application in cell and *in-vivo*, researchers modified the CRISPR-Cas9 system by integrating CRISPR RNAs (crRNAs) with manually designed trans-activating crRNA (tracrRNA) to form single guide RNAs (sgRNAs) (Jinek et al., 2012). SgRNAs can bind to complementary genomes, and the binding specificity is determined by the 20 nucleotides sequence before the three nucleotide protospacer adjacent motifs (PAM, composed of NGG or NAG sequences) in genomes (Jinek et al., 2012; Sternberg et al., 2014). The endonuclease Cas9 protein is guided to the target site by sgRNA, acting as a pair of “scissors” to cut DNA, leaving DSBs, single strand nick (Cong et al., 2013; Mali et al., 2013). Again, the host cell responds to DSBs through two different repair mechanisms: NHEJ and HDR. NHEJ is an error-prone repair mechanism that often leads to insertions or deletions (indels). These indels lead to frameshift mutations, premature stop codons, and/or nonsense mediated decay of target genes, leading to loss of function. In contrast, HDR was used to assist recombination of DNA donor templates to reconstruct cleaved DNA (Zhan et al., 2019). With guidance by sgRNA, specific genomic sites can be quickly located, leading to gene deletion, mutation, and insertion, making CRISPR a powerful research tool. Moreover, Specifically, with a single sgRNA, Cas9 can disrupt the open reading frame by inducing a frameshift mutation (Cox et al., 2015). While, using two sgRNAs targeting one chromosome, sequence deletions between two DSBs or the generation of chromosomal translocations can be achieved (Ghezraoui et al., 2014; Maddalo et al., 2014; Vanoli et al., 2017).

In addition to direct modification of genomic DNA, CRISPR can also be utilized to regulate the expression of target genes. The regulation of target gene expression is dependent on a nuclease-deficient Cas9 (dCas9), which is fused to a variety of effector domains to mediate specific local DNA manipulation (Qi et al., 2013). CRISPR activation (CRISPRa) and CRISPR inhibition (CRISPRi) are two important dCas9-based technologies. For instance, CRISPRa can be mediated by a dCas9 fused with the transcription activation domain VP64 to induce the expression of target genes (Gilbert et al., 2013). Furthermore, CRISPRa may fuse dCas9 with a tripartite activator like VP64-p65-Rta for improved efficiency (Chavez et al., 2015; Konermann et al., 2015). On contrary, CRISPRi is carried out by a dCas9 fusion with the Kruppel-associated cassette (KRAB) transcriptional repressor domain (Gilbert et al., 2014). As the superiority of CRISPR to previous gene editing technology (ZFNs, TALENS and RNA interferences), such as lower cost, easier manipulation, less time-consuming, high efficiency, low noise and limited off-target effects, being recognized, CRISPR technology has been widely used in basic research and quickly applied in translational studies.

Large scale gene screen tools based on the CRISPR-Cas9 system have been used to analyze gene functions and biological pathways related to human diseases (including cancer), which has become a revolution for research (Komor et al., 2017; Knott and Doudna, 2018). Eight years ago, several groups independently reported the use of the CRISPR-Cas9 library in human or mouse cells for large-scale knockout screens, identifying and analyzing functional molecules, ushering in the era of functional genomics research and establishing a new paradigm for the discovery of potential drug targets (Koike-Yusa et al., 2014; Shalem et al., 2014; Wang et al., 2014; Zhou et al., 2014). Since then, CRISPR screen technology has been rapidly developed to explore cancer treatment targets, including those related to tumor cell survival, proliferation, metastasis, synergistic lethality, drug resistance and immune evasion (Hou et al., 2017; Steinhart et al., 2017; Wang E. et al., 2019; Wang C. et al., 2019; Hinze et al., 2019).

Apart from basic researches, current clinical trials (Table 1) exploiting CRISPR-Cas9 technology mainly focus on improving the function of T cells, including generating CAR-T cells *in vitro* after collecting autologous T cells from patient, or activating T cells by knocking out PD-1 genes before infusion T cells to patients. For ethical and other reasons such as safety concern, it is not suitable for CRISPR-Cas9 technology to be directly applied in the human body so far, but *in vitro* modification of T cells such as CAR-T and TIL, followed by reinfusion therapy, can be achieved. Though, in preclinical studies, CRISPR technology has demonstrated encouraging result and efficacy in cancer treatment, multiple issues should be addressed before CRISPR-cas9 technology being used for human therapy, including off-target effects, immune responses elicited by Cas9 proteins, selection of target cells and so on. Once all these issues have been finally addressed, the CRISPR-cas9 technology will show large potential clinically in the various aspects not limited to cancer immune therapy and elimination of tumor cells.

**TABLE 1:** The ongoing clinical trials of immunotherapeutic agents that include a CRISPR/Cas9 element, as found in clinicaltrials.gov.

Disease	Country	Phase	Cell type	Target	Intervention	ID
Mesothelin Positive Multiple Solid Tumors, adult	China	I	Solid Tumors	mesothelin	Biological: anti-mesothelin CAR-T cells	NCT03545815
Mesothelin Positive Multiple Solid Tumors, adult	China	I	Solid Tumors	mesothelin	Mesothelin-directed CAR-T cells	NCT03747965
B-cell Malignancy,	United	I	B cell	CD19	Biological: CTX110	NCT04035434
Non-Hodgkin Lymphoma,	States					
B-cell Lymphoma,						
Adult B Cell ALL						
Esophageal Cancer	China		Solid Tumors		Other: PD-1 Knockout T Cells	NCT03081715
B-cell Lymphoma,	United States	I/II	B cell	CD19	Biological: CTX112	NCT05643742
Non-Hodgkin Lymphoma,						
B-cell Malignancy,						
Chronic Lymphocytic Leukemia (CLL)/Small Lymphocytic Lymphoma (SLL),						
Follicular Lymphoma,						
Mantle Cell Lymphoma,						
Marginal Zone Lymphoma,						
Large B-cell Lymphoma						
T Cell Lymphoma	United States	I	T cell	CD70	Biological: CTX130	NCT04502446
Clear Cell Renal,	United States	I/II	T cell	CD70	Biological: CTX131	NCT05795595
Cell Carcinoma,						
Cervical Carcinoma,						
Esophageal Carcinoma,						
Pancreatic Adenocarcinoma,						
Malignant Pleural Mesothelioma						
Metastatic Non-small Cell Lung Cancer	China	I	T cell		Drug: Drug: Cyclophosphamide	NCT02793856
					Other: PD-1 Knockout T Cells	
Multiple Myeloma	United States	I	B cell	BCMA	Biological: CTX120	NCT04244656
B Cell Leukemia,	China	I/II	B cell	CD19	Biological: UCART019	NCT03166878
B Cell Lymphoma						
Renal Cell Carcinoma	United States	I	T cell	CD70	Biological: CTX130	NCT04438083
B Cell Leukemia,	China	I/II	B cell	CD19,	Biological: Universal Dual Specificity CD19 and CD20 or CD22 CAR-T Cells	NCT03398967
B Cell Lymphoma				CD20,		
				CD22		
Advanced Hepatocellular Carcinoma	China	I			Biological: PD-1 knockout engineered T cells	NCT04417764

## 3 Application of CRISPR screen in cancer cells

**FIGURE 1:**
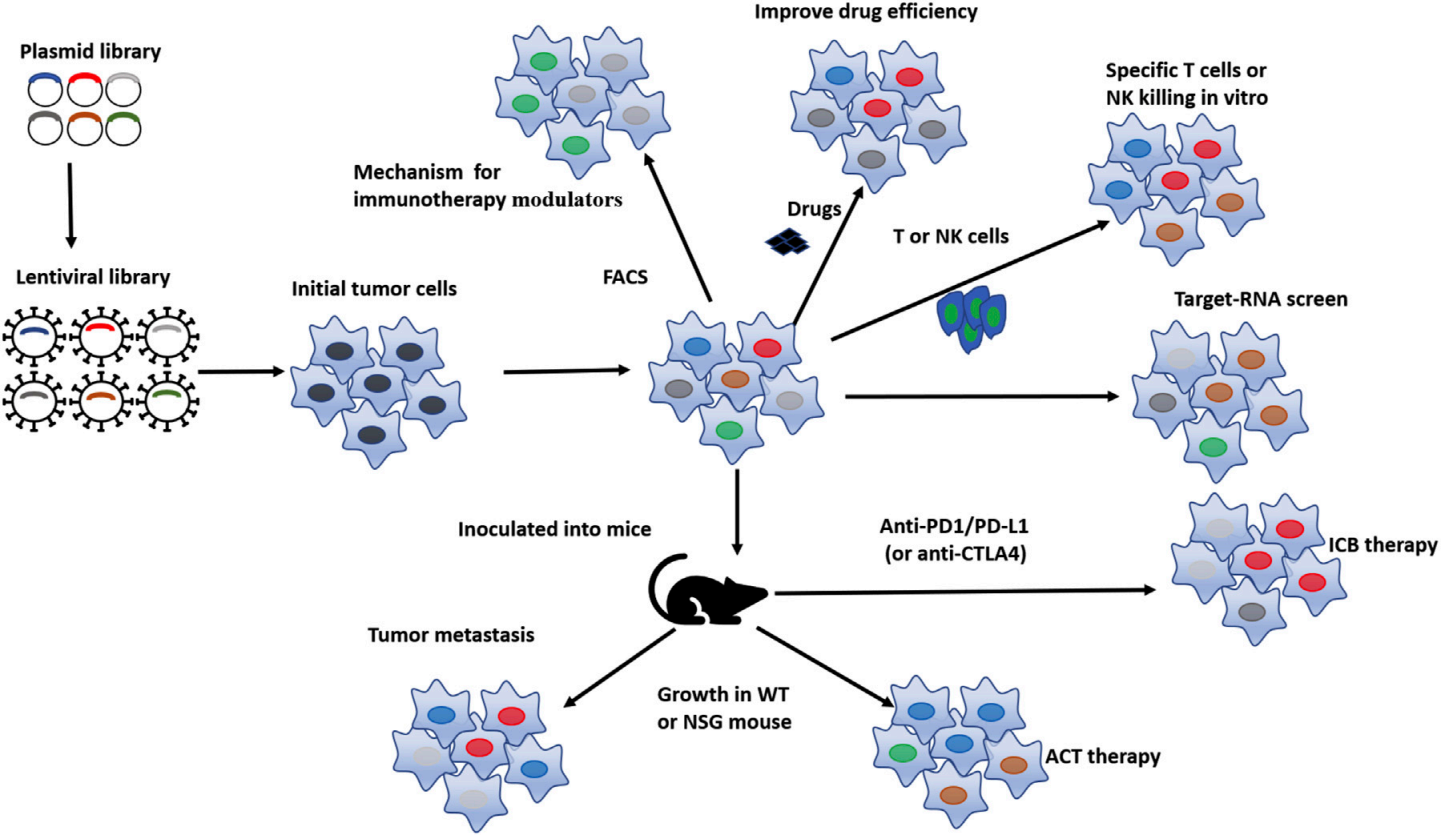
Pattern diagram of *in-vitro* and *in-vivo* CRISPR screens conducted on tumor cell. Based on the gene library of interested, lentivirus baring sgRNA towards these genes were designed and prepared, lentivirus were subsequently transduced to tumor cells and then the viral-transduced tumor cells were selected by resistance selection, in selected tumor cells, targeted genes were successfully processed by CRISPR system, then the obtained selected tumor cells are screened through different methods to obtain cell subsets of interest according to different demand, such as to improve ICB therapy efficacy, tumor sensitivity to drugs, T cell- or NK cell-killing *in-vitro*, as well as to reduce tumor metastasis, enhance tumor’s sensitivity to ACT therapy and ICB therapy *in vivo*. Finally, the differences were analyzed to identify potential functional genes.

The initiation and progression of cancer are related to the mutation and dysregulated expression of a series of genes, including oncogenes, tumor suppressor genes, T-cell or NK-cell killing escape genes, chemotherapy resistance genes, metabolism related genes, and cancer stem cell related genes (Zhang et al., 2021). The goal of cancer treatment is to inhibit tumor growth and progression, while correcting the specific mutations in tumor cells and reviving the inactivated genes are important means of tumor treatment. To identify genes essential for cancer initiation, progression, and drug escape, CRISPR screen technology has been widely used in basic cancer research and has made some quite exciting progress (Figure 1).

### 3.1 CRISPR screen for gene regulating tumor growth

CRISPR screen is a powerful tool in exploring critical genes that regulate tumor growth. To unveil the role of reactive oxygen species (ROS) in colitis and colon tumorigenesis, a genome-wide CRISPRko screen was used to systematically identify genetic factors involved in the regulation of oxidative stress. Next-generation sequencing (NGS) revealed that more than 600 sgRNAs, including those targeting LGALS2, were highly enriched in cells that survived under the sublethal H_2_O_2_ challenge. Further investigation demonstrated that LGALS2 inhibits H_2_O_2_-induced STAT3 phosphorylation and plays a suppressive role in colon tumor growth (Li H. et al., 2021). In another study illustrating the regulation of HCC growth under hypoxia, aldolase A (ALDOA), a key enzyme for glycolysis and gluconeogenesis is confirmed through genome-wide CRISPRko screen to be an important driver of HCC cells growth under hypoxia conditions. Knockout of ALDOA in liver cancer cells leads to lactate depletion, thereby inhibiting tumor growth (Niu et al., 2021). Notably, to explore the function of potential tumor driver genes for lung cancer, a genome-wide CRISPR screen was also performed in 2D monolayer of lung cancer cell and 3D lung-cancer spheroids in parallel. It is revealed that the CRISPR phenotype in 3D models recapitulated that of tumors *in vivo* more accurately, and genes with differential sensitivity in 2D and 3D conditions were highly enriched for those mutated in lung cancer *in vivo*, such as carboxypeptidase D (Han et al., 2020). In an effort to find potential targeted therapeutic drugs for lung squamous cell carcinoma (LUSC), MECOM was identified as a therapeutic target candidate for LUSC by a CRISPR-mediated screen based on 38 genes which were consistently normally amplified in three pairs of primary tumors and patient-derived xenografts (PDXs). High levels of MECOM expression are associated with a poor prognosis clinically. Consistently, enhanced expression of MECOM in LUSC cell lines was observed to promote the properties of cancer stem cells (CSCs), while knockout of it inhibits CSC phenotype (Ma et al., 2022).

### 3.2 CRISPR screen for genes modulating tumor metastasis

Metastasis is a leading cause of cancer patient death, because the metastatic tumors are usually drug-resistant and hard to remove through surgery (Lambert et al., 2017; Tasdogan et al., 2021). In metastasis, cancer cells first delaminate from their origins to invade the surrounding tissues, and then migrate to new sites through tissues, blood, and/or lymph system. As the initial dissociated cancer cells need to overcome a diversified and changing environment, only a few of them can survive in this process, and further grow in the metastatic sites (Vanharanta and Massague, 2013; Tasdogan et al., 2021). However, once the metastasis occurs, the survival of patients will be seriously threatened, and the treatment of tumors will also become more difficult (Kienast et al., 2010; Sela et al., 2021; Tasdogan et al., 2021).

The CRISPR screen is also a great tool to explore the mechanism of metastasis. A genome-wide CRISPRko screen was employed to identify genes regulating tumor growth and metastasis. When transplanted into immunocompromised mice, the edited cancer cell pool rapidly metastasized. The depletion of a small group of genes such as Nf2, Pten, and tripartite motif-containing protein 72 (Trim72) increased the metastasis in the lung. And the depletion of Cdkn2a, Fga, and Cryba4 accelerated the growth of primary tumors (Chen et al., 2015). Similarly, to identify genes that promote metastasis, an *in vivo* genome-wide CRISPRa screen was performed in circulating cancer cells from breast cancer patients. Ribosomal protein coding genes and translation regulators were enriched in the screen. Among them, RPL15 encodes a component of large ribosomal subunits, whose overexpression was found to increases the metastatic cell growth in multiple organs by selectively enhancing the translation of other ribosomal proteins and cell cycle regulators (Ebright et al., 2020). Epithelial plasticity, a reversible process regulate cellular epithelial and mesenchymal characteristics is related to tumor metastasis and chemotherapy resistance. In a CRISPRko screen for vital epigenetic genes that regulate, epithelial plasticity, the histone-modifying enzymes Zeb1 and Nsd2 were found to be involved in the writing and erasing of H3K36me2. Based on the screen, a unified epigenetic mechanism through which histone specific modifications regulated cell plasticity and metastasis in cancer cells was illustrated (Yuan et al., 2020).

### 3.3 CRISPR screen for gene affecting tumor drug efficacy

Every year, many drugs are approved by the U.S. Food and drug administration (FDA) for cancer treatment, and each approval indicates a step forward in fighting against cancer. However, the proportion of patients that benefit from drug therapy is still relatively low. Finding the mechanism of drug resistance and further developing of new drugs and potential combination therapy are one focus and major challenges of cancer research.

Synthetic lethality is defined as a phenomenon that simultaneous loss of two genes leads to cell death, but single deletion of either has little effect on cell viability (Huang A. et al., 2020). The concept of synthetic lethality in cancer has been extended to a pair of genes, where one gene is inactivated or over-activated and the expression of the other gene is reduced by drug treatment, resulting in cancer cell death, while normal cells (lack fixed gene changes) are protected from drug inhibition (Huang A. et al., 2020). In order to explore the reason for the limited therapeutic effect of sorafenib, Cun Wang and his colleagues conducted a synthetic lethal screening based on CRISPR-Cas9 to search for kinases that interacts with sorafenib. They confirmed that the inhibition of ERK2 (MAPK1) made some liver cancer cell lines sensitive to sorafenib (Wang et al., 2018). Similarly, CCNE1 is a commonly amplified gene in multiple tumor types, especially in high-grade serous ovarian cancer, uterine tumor and gastroesophageal cancer (Patch et al., 2015; Yuan et al., 2018; Watkins et al., 2020). To explore the therapeutic targets of tumors with CCNE1 amplification, David Gallo et al. did a genome-wide synthetic lethality CRISPRko screen. It is found that increasing CCNE1 dose results in vulnerability to inhibition of PKMYT1 kinase, a negative regulator of CDK1 (Gallo et al., 2022).

The widespread application of synthetic lethality in the mechanism of tumor treatment. A high proportion of NRAS mutations occur in melanoma patients. However, there has been limited progress in the development of targeted therapies for such patients. MEK inhibitors (MEKi) have shown certain clinical efficacy but need to be optimized. Weijia Cai et al. conducted a genome-wide CRISPRko screen, and found that deletion of phosphoinositide dependent kinase-1 (PDPK1) enhanced the efficacy of MEKi. The synergistic effect of PDPK1 deletion and MEKi is further validated in a melanoma cell line with the NRAS mutation using pharmacological and molecular methods (Cai et al., 2022). In another investigation, Lenvatinib is multi-receptor tyrosine kinase inhibitor that is used to treat patients with advanced HCC, but the ratio of patients respond to the drug is only 19% (Kudo et al., 2018). Haojie Jin et al. found that treatment of lenvatinib combined with inhibitor of epidermal growth factor receptor (EGFR) promotes the synthetic lethality of liver cancer cells in a CRISPRko screen with a kinome-focused library. The combination of EGFR inhibitors gefitinib and lenvatinib show potent anti-proliferative effects *in vitro* and *in vivo* (Jin et al., 2021).

CRISPRi knockdown technology suppresses gene expression by interfering with transcription rather than inducing double stranded DNA breaks (Gilbert et al., 2013; Qi et al., 2013; Gilbert et al., 2014). Compared with shRNA, another normal way to knockdown genome in cancer cells, CRISPRi causes low noise, minimal off-target effects and consistent activity across reagents (Zheng et al., 2018). The advantage of CRISPRi screen is that it is closer to the effect of a drug inhibitor and does not completely inhibit gene’s activity. Therefore, CRISPRi technology can distinguish the enzymatic effect of gene products from the non-pharmacologically inhibited scaffold effect. Yichen Xu et al. found that the RNA binding function of ERα is uncoupled with its DNA binding activity, and is crucial for the progression of breast cancer. Using genome-wide cross-linked immunoprecipitation (CLIP) sequencing and a functional CRISPRi screen, they confirmed that ERα-associated mRNA maintained the adaptability of cancer cells and triggerred the response of cells to stress through controlling RNA metabolism. In particular, they proved that the RNA binding of ERα mediated the selective splicing of XBP1 and the translation of eIF4G2 and MCL1 mRNA, which helped cancer cells to survive under stress conditions and maintain the tamoxifen resistance (Xu et al., 2021). However, the limited number and poorly characterization of knowntranscription start sites in genome has limited the application of CRISPRi and may introduce false negative results in large-scale screen (Huang A. et al., 2020). Collectively, the CRISPR screen tools are not only of great significance in exploring the mechanisms of existing tumor drugs, but also effective in finding their tolerance mechanisms, which will eventually contribute to solving problems of drug resistance and development of new drugs.

### 3.4 CRISPR screens for genes mediating immune evasion of tumors

#### 3.4.1 Screens for genes essential for tumor escape from CTL killing

Because CD8^+^ T cells are the most important tumor-killing cells in the solid tumor niche, immunotherapy mainly aims to improve the effector function of CD8^+^ T cells. Immunotherapy for tumors is divided into four major categories: immune checkpoint blocking (ICB) therapy, tumor vaccines (provenge, cimavax), adoptive T cell therapy (ACT, including TIL, TCR-T, and CAR-T therapy), and nonspecific immune modulators (Rosenberg et al., 1994; Cunningham et al., 2004; Bagley et al., 2018; Zheng et al., 2022).

CRISPRko screen is a powerful tool to explore critical genes in cancer cells that facilitate escape from T-cell killing. Deng Pan et al. performed a genome-wide CRISPRko screen and demonstrated on epigenetic mechanism explaining how cancer cells escape from CD8^+^ T cell-mediated killing (Pan et al., 2018). They found that depletion of *Pbrm1*, *Arid2*, *or Brd7*, which are all key components of the PBAF SWI/SNF chromatin remodeling complex, sensitized melanoma cells to T-cell killing. Loss of the PBAF components increase tumor cell sensitivity to IFNγ stimulation, enhance its chemokine secretion, and subsequent recruitment of effector T cells (Pan et al., 2018). Pancreatic ductal adenocarcinoma (PDA), another type of tumor, is characterized as inherently at immune suppressive status, Nina Frey et al. performed CRISPRko screens *in vitro* and *in vivo* to systematically analyze the intrinsic mechanism of PDA cells escaping from CD8^+^ T cell killing, identified Vps4b and Rnf31 as essential factors for tumor evasion from CD8^+^ T-cell killing (Frey et al., 2022). Loss of Vps4b in cancer cells impairs autophagy, leading to increased accumulation of Granzyme B intracellular, and thus enhances the sensitivity of tumor cell to CD8^+^ T cell effector function. They also found that Rnf31 protected cancer cells from TNF mediated caspase 8 cleavage and subsequent apoptosis induction, a mechanism that is conserved in human PDA-like organs (Frey et al., 2022). Min Li et al. performed genome-wide CRISPRko screen to identify genes involved in tumor escape from T cell-mediated killing, and identified that multiple IFNγ signaling-related genes were essential for the resistance of melanoma cells to T cell killing (Li M. et al., 2021). Moreover, they found deletion of the deubiquitinating protease ubiquitin specific peptidase 22 (USP22) in melanoma cells decreased the efficacy of T cell-mediated killing *in vitro* and *in vivo*, while USP22 overexpression enhanced tumor-cell sensitivity to T cell-mediated killing (Li M. et al., 2021).

To explore the core genes and signal pathways that help cancer cells to evade CTL-mediated killing, Keith A. Lawson et al. conducted genome-wide screens using mouse colorectal cancer, breast cancer, melanoma, and kidney cancer cell lines, with or without tumor-specific T cells, and identified 182 genes vital for cancer cell immune evasion. They found individually loss of these genes increased the sensitivity or resistance of cancer cells to CTL-mediated toxicity killing (Lawson et al., 2020).

#### 3.4.2 CRISPR screens for genes affecting tumor resistance against CAR-T cells

Chimeric antigen receptor (CAR) therapy is effective in the treatment of hematological malignancies, but has limited efficacy for solid tumors therapy (Maude et al., 2018; Cohen et al., 2019; Wang et al., 2020). The living environment of solid tumor, the tumor niche, may be inherently resistance against CAR-T therapy for complicated cellular mechanism, making it difficult for CAR-T cells to kill cancer cells (Bagley et al., 2018). In order to systematically identify potential resistance pathways in an unbiased manner, Rebecca C. Larson et al. conducted a genome-wide CRISPRko screen in glioblastoma (GBM). Through co-culturing tumor cells with CAR-T cells, they found that deletion of downstream genes in IFNγ signal pathway downstream genes such as *IFNGR1*, *JAK1*, *or JAK2* make GBM and other solid tumors more resistant to CAR-T cell killing *in vitro* and *in vivo*. However, the absence of gene in this pathway did not make leukemia or lymphoma cell lines insensitive to CAR-T cells (Larson et al., 2022).

#### 3.4.3 CRISPR screens for genes involved in tumor escape from NK-cell killing

Natural killer (NK) cells are unique group of congenital lymphocytes that is able to recognize, further remove virus infected cells or cancer cells. NK cells execute cytotoxicity function through multiple pathways with a variety of mechanisms, such as the production of cytokines, to regulate immune responses including anti-cancer immunity. To identify the key regulatory factors for tumor sensitivity or resistance against NK cell-mediated cytotoxicity in human glioblastoma stem cells (GSCs), Davide Bernareggi et al. performed a whole genome CRISPRko screen in GSCs and identify CHMP2A as a regulator of resistance to NK cell-mediated cytotoxicity. They found that the deletion of CHMP2A activated NF-κB in cancer cells and increased chemokine secretion, which subsequently promoted the migration of NK cells to cancer cells (Bernareggi et al., 2022). In order to systematically explore the sensitivity of human cancer cells to NK cells, Michal Sheffer et al. quantified reactivities of “DNA barcoded” solid tumor cell lines to NK cell and applied the CRISPR screen system to identify genes regulating cancer cell responses to NK cell killings. In these orthogonal studies, cancer cells sensitive to NK cells exhibit a “mesenchymal like” transcriptional program: high transcriptional characteristics of chromatin remodeling complex, enhanced expression of B7-H6 (NCR3LG1), and reduced expression of HLA-E/antigen presenting gene (Sheffer et al., 2021). In another study, Conor J Kearney et al. conducted a series of CRISPR screens to explore the mechanism of cancer cells’ escaping from CD8^+^ T cells and NK cells killing. It is found that loss of key genes in TNF signaling, IFN-γ signaling or antigen presentation pathway could enhance cancer cell resistance to CD8^+^ T cell-mediated killing and weaken the effect of anti-tumor immune response *in vivo*. The deletion of downstream genes in the TNF pathway promotes the cancer cells to escape from the killing by primary NK cells. They also determined that the metabolic protein 2-aminoethanethiol dioxygenase (Ado) regulated the sensitivity of cancer cells to TNF-mediated killing by cytotoxic lymphocytes, which is necessary for optimal tumor control *in vivo* (Kearney et al., 2018).

#### 3.4.4 CRISPR screens for genes involved in tumor resistance against ICB therapy

Immune checkpoint factors are co-inhibitors of effector lymphocytes, which can reduce the activation of lymphocytes and prevent their overactivation. Cancer uses this physiological mechanism to evade the anti-tumor immune response by expressing corresponding ligands in cancer cells, stromal cells or exosomes (Chen and Flies, 2013; Daassi et al., 2020). Co-inhibitory receptors include CTLA4, PD1, T-cell immunoglobulin and mucin domain containing-3 (TIM3), lymphocyte-activation gene 3 (LAG3) and so on. Among them, CTLA4 is expressed by activated T cells, mainly Treg cells, to prevent the activation of effector T-cell. PD1 is expressed by activated T cells, NK cells and other cells like Treg cells, MDSCs, monocytes, and dendritic cells (DC), while its ligand PDL1 is expressed by many stromal cells and cancer cells, as well as myeloid including DCs (Jain et al., 2010; Sangro et al., 2021). The major obstacle for improving efficacy of ICB therapy is that the obscure and complicated mechanism for cancer cell escaping from T cell killing. Thus, exploring the mechanisms explaining how tumor cells escape immune surveillance, and designing appropriate combination drugs of ICB therapy are the current focuses of tumor immunology research. The CRISPR screen system is a powerful tool to solve these questions.

Robert T. Manguso et al. employed the genome-wide CRISPRko screen to identify genes that regulate immunotherapy of mouse melanoma, and found that deletion of genes involved in several pathways sensitized the tumor to anti-PD-1 blockade therapy. *Ptpn2*, which encodes protein tyrosine phosphatase, is found to negatively regulate IFNγ-mediated effects on antigen presentation and anti-PD-1 blockade therapy. In addition, previously known genes including *pd-l1*, *cd47*, *Stat1*, *Jak1*, *Ifngr2*, *Ifngr1*, and *Jak2* have been confirmed for their roles in immune escape (Manguso et al., 2017). Although immunotherapy has made substantial progress in the treatment of lung adenocarcinoma (LUAD), the overall response rate of patients with KRAS mutant LUAD is still low. Fei Li et al. employed an *in vivo* CRISPR screen with an epigenetic library in the KrasG12D/Trp53^−/−^ LUAD model to identify epigenetic regulators of tumor immunity and discovered that loss of histone chaperone Asf1a made cancers sensitive to anti-PD-1 therapy. Their results provided a theoretical basis for a new combination therapy consisting of Asf1a inhibition and anti PD-1 immunotherapy (Li et al., 2020). Immunotherapy has deeply changed cancer treatment, but only a few patients with pancreatic ductal adenocarcinoma (PDA) can benefit from it, which is mainly due to the poor infiltration and inactivation of T cells in the tumor microenvironment (TME). Jinyang Li et al. did an *in vivo* CRISPR screen and determined that lysine demethylase 3A (KDM3A) is an effective epigenetic regulator of the PDA immunotherapy response. In mechanism, KDM3A acts through Krueppel like factor 5 (KLF5) and SMAD family member 4 (SMAD4) to regulate the expression of epidermal growth factor receptor (EGFR) (Li J. et al., 2021). In order to find out the mechanism of the limited efficacy of ICB therapy for triple negative breast cancer (TNBC), Xiaoqing Wang et al. conducted an *in vivo* CRISPRko screen in the syngeneic TNBC mouse model and confirmed that the deletion of E3 ubiquitin ligase Cop1 in TNBC cells reduced the macrophage infiltration and the secretion of chemokines, led to enhancing anti-tumor immunity and improving the efficacy of ICB therapy for tumor (Wang X. et al., 2021). These data shown that the tool of screen based on CRISPR-Cas9 technology is of great significance in exploring the mechanism of T cell killing.

The inactivation of IFNγ signal pathway is the key for cancer cells to escape CAR-T cell killing, T cell killing, and ICB therapy (Manguso et al., 2017; Pan et al., 2018; Larson et al., 2022). Further digging into the genes regulated by the IFNγ pathway is not only of great significance for the basic research of tumor immunity but also important for the treatment of clinical patients.

#### 3.4.5 CRISPR screen for mechanisms of critical modulators in immunotherapy

The Fluorescence Activating Cell Sorter (FACS) is a powerful tool to examine membrane and intracellular protein expression, FACS-dependent CRISPR screen is a method useful tool for study the mechanisms of critical modulators in immunotherapy. Lots of cell membrane proteins play key roles in the process of tumorigenesis, progression, and metastasis. They are receptors, activation, inhibition or escape related proteins. To study function and modulation of such membrane proteins, FACS-dependent CRISPR screen is a powerful technology and has significant contribution to basic and clinical cancer research, such as those investigating unknown modulators for current immune therapy. PD-L1 is one of the important targets of tumor immunotherapy, and the expression of PD-L1 on the surface of cancer cells can be easily detected by flow cytometry. Marian L. Burr et al. applied a genome-wide CRISPRko screen to prove that in a serious of tumors, CKLF like MARVEL transmembrane domain of protein 6 (CMTM6) is a critical regulator of PD-L1 expression. In various the *in vivo* and *in vitro* experiments with these cancer cells, depletion of CMTM6 reduced the expression of PD-L1 and thus relieved the tumor-specific T cells from inhibition state (Burr et al., 2017; Mezzadra et al., 2017).

The presentation of tumor associated antigens by MHC class I molecules is a prerequisite for effective antitumor CD8^+^ T cell response. The reduced MHC-I expression is a common mechanism for tumor immune escape. Lotte Spel et al. identified Nedd4 binding protein 1 (N4BP1) and TNFAIP3 interacting protein 1 (TNIP1) as NF-κB-dependent MHC-I inhibitors, through a FACS-dependent genome-wide CRISPRko screen in neuroblastoma. The loss of N4BP1 or TNFAIP3 promotes the expression of MHC-I on tumor cell membranes, thus enhances T cell recognition and CD8^+^ T-cell activation (Spel et al., 2018). CD47 is widely and highly-expressed in cancer cells and is one of the inhibitory ligands of myeloid cells. Blocking CD47 and its receptor signal regulatory protein-α (SIRPα) can enhance the phagocytosis of macrophages or neutrophils to destroy cancer cells (Jaiswal et al., 2009; Majeti et al., 2009). In order to identify potentially regulator of CD47, Zhiqiang Wu et al. applied a FACS-based genome-wide screen on HCT116 human colon cancer cells, and found glutamine peptide cyclotransferase like protein (QPCTL) is a key regulator of CD47 (Wu et al., 2019). Another study by Meike E. W. Logtenberg et al. almost simultaneously identified that QPCTL plays a vital role in regulating CD47-SIRPα signaling in checkpoint block through haploid genetic screen (Logtenberg et al., 2019). All these researches implied the feasibility of applying FACS-dependent CRISPR screen in studies of cell membrane proteins.

### 3.5 CRISPR screen targeting non-coding RNA

At the beginning, non-coding RNA (ncRNA) is considered as selfish RNA, and its function in the organism is not clear. As research continues, severe mutations in non-coding RNA regions of the human genome have been found as implications for cancer risk (Zhang and Meyerson, 2020). This is not surprising, actually, the non-coding region contains a variety of functional elements that regulate oncogenes, tumor suppressors and related genes (Zhang and Meyerson, 2020). Long non-coding RNAs (LncRNAs) have now been demonstrated to play an important role in gene regulation in normal and cancer cells, including regulating gene activation and silencing, X chromosome inactivation, selective expression and post-translational modification (Liu et al., 2021). Several ncRNA targeted cancer drugs are currently in clinical trials, such as MRX34, a microRNA 34a (miR-34a) mimic and cobomarsen, a miRNA-155 inhibitor (Seto et al., 2018; Hong et al., 2020). However, the functions of ncRNA in tumorigenesis, development and therapy are still misty. Recently, the addition of CRISPR-based genome-wide knockout screen and transcriptome engineering toolbox has enabled researchers to better understand how ncRNAs interfere with the cancer phenotype.

Several groups used pooled saturation mutagenesis CRISPR nuclease screens to identify the essential cis-regulatory element of one or more genes (Canver et al., 2015; Sanjana et al., 2016). A CRISPR screen employing ∼18,000 sgRNAs which targeted >700 kb regions surrounding the genes *NF1*, *NF2*, and *CUL3* was developed to search for resistance to BRAF inhibitor in melanoma (Sanjana et al., 2016). These noncoding locations that modulate drug resistance also harbor predictive hallmarks of noncoding function, such as modulation of transcription factor occupancy and long-range or local epigenetic environment (Sanjana et al., 2016). In addition to exploring gene enhancers for cancer resistance, CRISPR screen targeting transcription factor binding sites were also used to study transcriptional regulation of some known factors. Agami and colleagues focused on the binding site of two transcription factors-p53 and estrogen receptor α (ERα), both of which have definite roles in cancer. Using two independent CRISPR-Cas9 screens, they discovered a large number of enhancers required for p53-induced senescence and ER-regulated growth of breast cancer cells (Korkmaz et al., 2016). Charles P Fulco et al. reported similar screen of CRISPR-dCas9^KRAB^ inhibition (Fulco et al., 2016). Instead of focusing on DHS DNase I hypersensitive sites, they extended sgRNA targets to the entire genome. The results show complex relationships between genes and enhancers, including multiple genes controlled by one enhancer or multiple enhancers controlling a single gene. There is also evidence that enhancers compete with adjacent promoters in gene regulation (Fulco et al., 2016).

In order to determine genes and pathways that affect cancer cell sensitivity to cytarabine, the main drug for the treatment of acute myeloid leukemia (AML), Assaf C. Bester et al. created a genome-wide comprehensive platform based on integrated CRISPRa screen for both protein-coding and non-coding genes (Bester et al., 2018). Preliminary drug resistance genes were identified using pharmacogenetic data from 760 human pancreatic cancer cell lines. Subsequently, the genome scale function of coding region and lncRNA was characterized by CRISPR activation. For the evaluation of lncRNA function, they developed a CRISPR activation strategy targeting 14701 lncRNA genes. Cell cycle, survival/apoptosis and cancer signaling genes were identified by calculation and functional analysis. In their analysis, the transcriptional activation of GAS6-AS2 lncRNA led to the overactivation of the GAS6/TAM pathway, which is the drug resistance mechanism of many cancers, including AML (Bester et al., 2018). The team of Feng Zhang combined the dCas9-VP64 protein with an MS2-p65-HSF1 fusion protein to form the SAM complex, which can upregulate coding genes, non-coding RNA, and simultaneously activate multiple genes. The activation target depends on the design of sgRNA library, for example, sgRNA targeting more than 10000 lncRNA transcription start sites was designed and verified in melanoma cell lines (Joung et al., 2017).

Regardless of chemotherapy, targeted therapy, ICB therapy, or ACT therapy, the purpose of tumor therapy is to kill tumors or inhibit their proliferation and metastasis. Tumors escape treatment in many ways, such as epigenetic modifications, gene expression modifications, or extracellular secretion. In HCC patients, tumor cells escape levatinib treatment through increased expression of EGFR (Jin et al., 2021). The tumor cells in patients with metastatic melanoma or prostate cancer antagonize the effect of ICB treatment through the secretion of PD-L1 exosomes (Chen et al., 2018; Poggio et al., 2019). The CRISPR screen genome editing technology plays an important role not only in exploring the mechanism of tumor escape but also in finding potential therapy drugs. All in all, a tumor-target CRISPR screen may be one of the most useful tools to explore the mechanisms and therapy of tumors.

## 4 CRISPR screen for genes modulating immune cells activity

**FIGURE 2:**
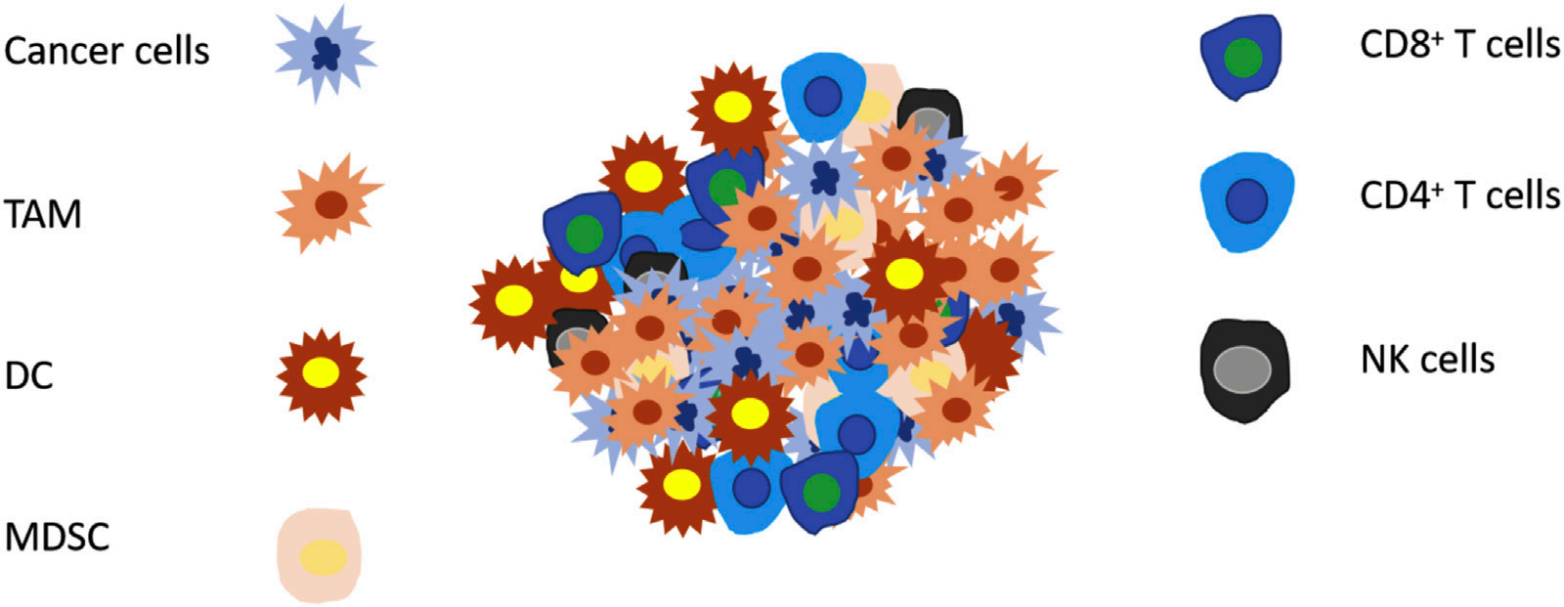
Common patterns of immune cells in tumor microenvironment. There are multiple subsets of immune cells in the tumor microenvironment, among which CD8 T cells, mature DC cells, NK cells, and Th1 cells promote the killing of tumors, while cells such as Treg cells, MDSC, TAM, and immature DC cells promote the escape of tumors. Up to now, there has been CRISPR screening based on CD8 T cells, Treg, and macrophages in tumor treatment, all of which provide some help for tumor treatment.

In the process of tumorigenesis and development, the activation of proto-oncogenes and the inactivation of tumor suppressor genes are two basic mechanisms for tumor occurrence. Further, in solid tumors, inhibition of killer cell’s function in the tumor microenvironment and the enhancement of immunosuppressive cell’s activities can both promote tumor progression. Moreover, immune responses in the tumor microenvironment may also participates in the tumor pathogenesis. For example, HCC is a typical inflammation-related cancer. About 90% of the HCC burden is related to persistent inflammation caused by viral hepatitis, excessive drinking, and nonalcoholic steatohepatitis (Villanueva, 2019). Thus, the tumor niche, the major site where inflammation occur, plays a key role in the pathogenesis of HCC (Ringelhan et al., 2018). Notably, exploring the function of immune cell subsets in tumor therapy is of great significance for clinical tumor therapy. ACT therapy (including CAR-T therapy, TIL therapy) and ICB therapy are based on the tumor killing CD8^+^ T cells in the immune microenvironment. Up to now, despite extensive application of single cell sequencing and other technologies has facilitated the classification of cell subsets in the tumor microenvironment, the exact function of each cell subset needs further investigation. The gene screen based on CRISPR-Cas9 technology provides a guarantee for in-depth and comprehensive studies (Figure 2).

### 4.1 CRISPR screen in T cells

Large-scale CRISPR screen in primary cells has always been difficult, and T cell screen also goes through a process from simple and rough to complex and deep. When in the progression of altering to exhausted status, CD8^+^ T cells have great changes in signal regulation, surface markers, and transcription factor expression (Philip and Schietinger, 2022), making studies on CD8^+^ T cells even more complicated. The aim of screens in T cells mainly focus on identifying key modulators of T cell functions, which can finally provide clues to enhance anti-tumor function of T cells from various aspects. Eric Shifrit et al. developed a method combining single guided RNA (sgRNA) lentivirus infection and Cas9 protein electroporation (SLICE), to identify regulators of primary human T cell stimulation response. Genome-wide loss-of-function screen identified important T cell receptor signaling components and genes that negatively regulate proliferation after stimulation. The characteristics of T cell mutations that enhanced killing activities towards cancer cells were verified by targeted knockout of a single candidate gene (Shifrut et al., 2018). Based on the key role of CD8^+^ T cells in the anti-tumor immune responses, Sidi Chen group directly conducted genome scale CRISPR screen on CD8^+^ T cells in the context of cancer immunotherapy, and determined the key factors regulating tumor invasion and degranulation. *In vivo* screen effectively reidentified typical immunotherapeutic targets, such as PD-1 and Tim-3. Infiltration and degranulation screen both identified RNA helicase Dhx37. Dhx37-knockout enhanced the efficacy of antigen-specific CD8^+^ T cells against triple negative breast cancer *in vivo* (Dong et al., 2019). In order to facilitate the identification of T cell targets, Sidi Chen’s group has also developed a hybrid genetic screen system that combines Sleeping Beauty (SB) transposons and single guided RNA cassettes by nesting them in adeno-associated viruses (AAV). They performed *in vivo* AAV-SB CRISPR screen with membrane protein targets from CD8^+^ T cells in a mouse model of GBM. Adoptive transfer of CD8^+^ T cells with edited PDIA3, MGA5, Emp1, or Lag3 improve the survival rate of GBM tumor-bearing mice in both syngeneic and T-cell receptor transgenic models (Ye et al., 2019).

Memory T (Tmem) cells maintain the stemness of T cells and play an important role in immunization and anti-tumor therapy. During the first division of activated CD8^+^ T cells, cBAF and MYC often distribute in two daughter cells asymmetrically, the ones with higher concentration of MYC and cBAF complexes differentiate to Teff cells, while others with less MYC and cBAF components preferentially differentiate to Tmem cells (Guo et al., 2022). Through a CRISPR-based screen *in vivo*, Ao Guo et al. obtained several components of mammalian typical BRG1/BRM related factors (cBAF) as negative regulators of Tmem cells and confirmed that cBAF complexes were essential for activated CD8^+^ T cells to differentiate into T effect (Teff) cells, and their knockout promoted the formation of Tmem cells (Guo et al., 2022). To investigate the molecular mechanism of T cell exhaustion, Julia A. Belk and his colleagues applied chronic stimulation test and carried out the whole genome screen both in tumor and T cells based on CRISPR-Cas9 genome editing technology to systematically search for the regulatory factors of T-cell exhaustion. In mouse and human tumor models it’s showed that disturbance of INO80 and BAF chromatin remodeling complexes improved T cell persistence in tumors. Subsequent Pertub-seq (scRNAseq after CRISPR) revealed different transcriptional effects for each complex. The deletion of typical BAF complex members (including Arid1a) led to the maintenance of effector programs and the downregulation of exhaustion-related genes in tumor infiltrating T cells (Belk et al., 2022). To explore the role of metabolism in regulating the process of early T cell differentiation, using *in vivo* CRISPRko screen, Hongling Huang et al. systematically analyzed metabolic factors in fate determination of Teff and Tmem, mainly focusing on negative regulatory factors related to Tmem, and found that amino acid transporters Slc7a1 and Slc38a2 partially inhibit the differentiation of Tmem by regulating mTORC1 signaling (Huang et al., 2021). The main goal of immunotherapy is to improve the effector activity of tumor antigen specific T cells. Although several Teff - driven transcription factors (TF) have been identified, little is known about the transcriptional coordination of Teff biology. Zeyu Chen et al. developed a CRISPR screen platform for T cells *in vivo*, and identified a mechanism suppressing Teff biology through the ETS family TF, Fli 1. They found that Fli1 inhibits the Teff genes and deletion of the *Fli1* gene enhances the Teff response without interfering memory or exhausted precursors (Chen et al., 2021).

### 4.2 CRISPR screen in CAR-T cells

CAR-T therapy is one of the most important clinical applications of immunotherapy, but it has been criticized for being less effective in solid tumors and expensive. Lower therapeutic effect, limited tumor types suitable for its application, and difficulties in constructing universal CAR-T are all obstacles restricting its development. The application of CRISPR-Cas9 gene editing technology may provide opportunities to overcome these obstacles. In order to find genes that can improve the therapeutic effect of CAR-T therapy, Lupeng Ye et al. developed a CRISPR activation screen based on dCas9 in primary CD8^+^ T cells and identified gain-of-function targets for CAR-T engineering. Subsequent knock-in or overexpression of PRODH2-the leading target, enhanced the killing effect and *in vivo* efficacy of CAR-T in many cancer models, probably due to the transcriptomics and metabonomics broadly reshaped in PRODH2 high expressioned CAR-T cells (Ye et al., 2022). To search for genes that enhance CAR-T killing of solid tumors and mechanisms by which solid tumors escape CAR-T killing, Dongrui Wang and his colleagues studied the molecular mechanism underlying CAR-T cells mediated killing of GBM through genome-wide CRISPR screen of both CAR-T cells and patient-derived GSCs. The screen of CAR-T cells identified effect genes, including TLE4 and IKZF2, knockout of these genes enhances the anti-tumor effect of CAR-T cells. Bulk and scRNA-seq of edited CAR-T cells showed superior effector function and a transcriptional profile of inhibition of an exhausted response. The screen of GSCs identified genes crucial to CAR-T mediated killing sensitivity, including RELA and NPLOC4, whose knockout changed tumor immune signals and improved the responsiveness of CAR-T therapy. In general, the screen based on CRISPR of CAR-T cells and GSCs has found ways to improve the efficacy of CAR-T cells to GBM, which may be extended to other solid tumors (Wang D. et al., 2021). To better understand the mechanisms influencing CAR-T cell cytotoxicity and improve the potential regulatory effect of current immunotherapy with small molecule drugs, Olli Dufva et al. systematically studied the available drug mechanisms of CAR-T cytotoxicity using more than 500 small molecules and genome-wide CRISPR-Cas9ko screen. Several tyrosine kinase inhibitors were found to inhibit the cytotoxicity of CAR-T cells by destroying T-cell signaling transcriptional activity. SMAC, on the other hand, mimics sensitized-B cell acute lymphoblastic leukemia and causes large B-cell lymphoma cells to diffuse to anti-CD19 CAR-T cells. CRISPR screen data confirmed that FADD and TNFRSF10B (TRAIL-R2) death receptor signals were the critical mediators of CAR-T cytotoxicity (Dufva et al., 2020).

### 4.3 CRISPR screen in NK cells

NK cells have strong cytotoxicity. After forming immune synapses with target cells, NK cells can trigger effective reactions by releasing cytolytic particles and cytotoxic cytokines, so NK cells play an important role in the anti-tumor immune response (Prager and Watzl, 2019). However, up to now, results of the CRISPR screen in NK cells are few, except one in the NK cell line NK92 cells. In order to improve the cytotoxicity of NK92, Rih Sheng Huang et al. verified multiple knockouts of activating or inhibiting receptors, and found that endogenous CD16 and DNAM-1 were reactivated by Cas9-mediated promoter insertion. The NK-92 with CD16 and DNAM-1 re-activated shows significantly enhanced cytotoxicity, and can mediate antibody dependent cytotoxicity to fight against cancer cell lines which are difficult to kill (Huang R. S. et al., 2020).

### 4.4 CRISPR screen in macrophage

Tumor associated macrophages (TAM) are closely related to cancer metabolism, malignant progression and drug resistance. More and more evidences show that TAM (mainly M2) can be reprogrammed into anti-tumor M1 macrophages (DeNardo and Ruffell, 2019). For example, CpG oligonucleotides can activate TLR9, which significantly enhances the anti-tumor activity of macrophage (Liu et al., 2019). Latest progress in this field has attracted great attention, showing that activating anti-tumor function of macrophages is a promising strategy for cancer immunotherapy. In the past few years, activation of inflammatory bodies, phagocytosis, and cell death has been determined as key regulators related to the biological behavior of macrophages using CRISPR screens (Napier et al., 2016; Jeng et al., 2019; Xu et al., 2019).

To explore the mechanism of cancer cells escaping phagocytosis. Roarke A. Kamber and his colleagues developed a platform to use complementary genome-wide CRISPRko and overexpression screen both in cancer cells and macrophages. In addition to known factors such as CD47, they have identified many ADCP sensitive regulators in cancer cells, including adipocyte plasma membrane associated protein (APMAP) enzymes which was poorly characterized. They further found that the deletion of APMAP combined with tumor antigen targeted monoclonal antibodies and/or CD47 blocking antibodies, significantly increase phagocytosis in a wide range of cancer cell types. Using the whole genome screen of macrophages, they found that the G-protein coupled receptor GPR84 enhanced phagocytosis of APMAP-deficient cancer cells. This work revealed the cancer internal regulator sensitive to antibody driven phagocytosis, and more broadly, expanded the understanding of the cancer resistance mechanism to macrophage phagocytosis (Kamber et al., 2021).

There are few CRISPR screen studies based on NK cells and MDSC cells. On one hand, primary cell culture is difficult, on the other hand, gene delivery to these cells through virus transduction or other methods are also not easy. Optimizing culture methods of primary cell subsets from tumor microenvironment and further study their functions and regulatory mechanisms is of great significance to expand applications of CRISPR in future.

## 5 Discussion and conclusion

CRISPR-Cas9 technology, as a stable, efficient, simple, and widely used genome editing technology, has only been available for about 10 years. However, CRISPR-Cas9 has made fundamental changes in research of agriculture, biotechnology, biomedicine, and other fields, but within no field has it had a more profound impact than cancer research, as the evidence by faster growth of publications. The use of genome wide knockout-, knockdown- or activation-screen made quickly and accurately discovery revealing new and more detailed mechanisms for people to better understand tumor growth, metastasis, and treatment. More importantly, CRISPR-Cas9 based screens have great potential in cancer therapy. It is not only possible to investigate the mechanisms of synergistic lethality and clinical drug tolerance, but it may also lead to the identification of new targets for their combined therapy. The technology is also used to screen T cells, DC cells, or macrophages to find potential therapeutic drugs or targets for ICB therapy and CAR-T therapy.

There is no doubt that CRISPR-Cas9 gene editing technology has obvious limitations. First of all, the biggest concern is the side-effects. At present, improving the specificity of targeting is not only important for basic research but also essential for its application. Secondly, another source of concern is on-target mutagenesis. In theory, CRISPR-Cas9 gene editing only results in cutting of double-stranded DNA, the terminal repairs of broken DNA are mediated by host and have great uncertainty. The deletion of large segments may affect the function of the whole genome, while the insertion of large segments, small fragments, and even frameshift mutations possibly produce novel proteins with unknown functions. Thirdly, the safety and effectiveness of the *in vivo* CRISPR system are also of concern. In the experimental model of tumor-bearing mice, the expression of the Cas9 protein will affect the growth of cancer cells *in vivo*. The tolerance of humanized acquired Cas9 protein can reduce the effectiveness of the CRISPR system *in vivo*. Fourthly, DNA double strand breaks caused by the CRISPR system can promote the activation of the P53 signal, thus initiate DNA damage repair and block the cell cycle, and further mediate cell death. However, careful design of sgRNA and systematic analysis can effectively reduce the activation of P53 protein, and a single Cas9 nickase approach can effectively avoid breakage of double stranded DNA (Enache et al., 2020; Zhang et al., 2021). Fifthly, mouse cancer cells with Cas9 overexpression have certain differences from the control cells in growth and tumor-bearing experiments, which may implicate the influence of Cas9 regulation on other genes expressed by cancer cells. Exploration of potentially regulated genes is critical for basic research and clinical applications of CRISPR technology. Finally, while the CRISPR system has higher editing efficiency in cancer cells, it exhibits lower editing efficiency in primary cells such as T cells and DC cells, while its application in MDSCs is rarely reported. Enhancing efficiency of gene delivery to primary cells and improving cell culture methods to keep activated or viable status of primary cells are both of greatly importance for the application of the CRISPR system in cancer research.

In spite of current limitations, powerful functions of CRISPR-Cas9 have been widely recognized soon after its first application. CRISPR technology has been widely used in various studies, from the initial single gene knockout to the genome-wide screen, from the knockout screen to the knockdown or overexpression screen, from the genome-wide screen to the screen of functional libraries with more specific purposes, and from the screen of cancer cells that are easy to culture to the primary T cells, DC cells, and macrophages that are difficult to culture. Recently, CRISPR-Cas9 technology, combined with single-cell RNA-seq, RNA-seq, ATAC-seq, CITE-seq, and other technologies, provides new opportunities and brings evolutions for tumor research. For example, scRNA-seq combined with CRISPR screen technology, single-cell CRISPR screen. analyze the changes in single-cell transcription caused by each sgRNA mutation, making it possible to screen target genes accurately and achieve each perturbed transcriptome simultaneously in high throughput ways.

In summary, the application of CRISPR screening has extensively improved the progress of cancer research in the past decade, unraveling many previously untouched mechanisms related to tumor proliferation, metastasis, and treatment (including Chemotherapy, immunotherapy represented by ICB therapy and CAR-T therapy), resulting in certain improvements in tumor prediction, diagnosis, and personalized treatment. The combination of CRISPR screen and other new technologies is of great significance in basic research and clinical treatment of cancer, hoping to play a key role in the transformation of tumors into controllable chronic diseases, and finally benefits patient in future.
